# Risk assessment of civil aviation cabin safety incidents based on the CNN–LSTM–Attention model

**DOI:** 10.1371/journal.pone.0343678

**Published:** 2026-05-27

**Authors:** Lianbin Zhou, Xi Wang, Weiwei Jiang, Peiwen Zhang

**Affiliations:** 1 School of Economics and Management, Civil Aviation Flight University of China, Guanghan, China; 2 Sichuan Dadu River Shuangjiangkou Hydropower Development Co., Ltd., Barkam, Sichuan, China; 3 Chengdu Low Altitude Economy High Quality Development Research Center, Chengdu, China; Cardiff Metropolitan University - Llandaff Campus: Cardiff Metropolitan University, UNITED KINGDOM OF GREAT BRITAIN AND NORTHERN IRELAND

## Abstract

Against the backdrop of the thriving global civil aviation industry, cabin safety, which is a crucial link in civil aviation safety systems, has prompted an increasingly urgent need for intelligent risk management. This study constructs an integrated framework of “deep text mining–intelligent risk assessment” and proposes a hybrid CNN-LSTM-Attention model for cabin safety incident classification. Using 8,280 global cabin abnormal event reports from 2004–2024, the model achieves 95.01% accuracy and an F1 score of 94.17% on the test set, substantially outperforming benchmark approaches. Compared with XGBoost (89.45% accuracy, 88.98% F1), the proposed model improves accuracy by 5.56% and the F1 score by 5.19%, demonstrating superior capability in extracting deep semantic features and identifying key risk patterns. The framework also establishes a “text feature–risk mechanism-hazard level” mapping system, enabling more interpretable risk quantification. The findings provide an effective technical path for intelligent early warning of cabin safety risks and offer methodological support for data-driven decision-making in civil aviation safety management.

## 1 Introduction

Cabin safety is the most direct and important component of flight safety. Cabin safety not only serves as a crucial foundation for increasing flight safety but also acts as the final barrier of the civil aviation safety transportation management system. With the rapid development of the global civil aviation industry, various large aircraft have been put into operation, rendering the cabin environment increasingly complex. The sources and composition of passengers have become more diverse, highlighting the difficulty and intensity of cabin safety management and cabin service management. Recently, cabin safety incidents have frequently occurred. The issues related to civil aviation cabin safety are intricate, and one important characteristic is that safety accidents have some degree of contingency and inevitability. Data from the International Civil Aviation Organization (ICAO) show that in 2023, global aviation safety presented a contradictory situation in which the accident rate decreased but the total number of accidents increased. The increase in flight volume has resulted in an increase in the absolute value of safety risk. Cabin safety incidents have become the primary type of abnormal civil aviation events (accounting for 41%), with Europe and the United States accounting for more than 50%. On average, one incident occurs per 800 flights, 12% of which result in flight delays or diversions, causing an annual economic loss of 280 million US dollars. Therefore, improving cabin safety risk management is extremely urgent.

Research on aviation safety trends focuses on the use of natural language processing (NLP) and machine learning technologies to explore in depth the value of aviation safety text data. Rose et al. [1] classified Aviation Safety Reporting System (ASRS) data via NLP-based clustering and visualization methods and combined K-means clustering with T-SNE visualization to automatically identify patterns and trends in aviation incident reports. This method can reveal safety hazards that traditional anomaly classification fails to detect [1]. Kuhn [2] analyzed ASRS data via structural topic modeling (STM) and reported that STM can reveal potential patterns in text data without expert annotations. In terms of aviation accident anomaly detection, research methods are gradually shifting from traditional models to efficient machine learning algorithms, with a focus on improving the real-time performance, accuracy, and early warning capabilities of detection. For example, Zhao et al. [3] further developed an incremental detection method based on the Gaussian mixture model (GMM), which can continuously adapt to new data without retraining, significantly improving the timeliness of flight data anomaly detection. Hassan and Granelli [4] use a one-dimensional Convolutional Neural Network (1D-CNN) deep learning mode to capture spatial relationships along the beamsteering angles to accurately predict changes in power in a dynamic channel, which are essential for optimizing beam selection and enhancing communication performance. Chevrot et al. [5] designed a discriminative autoencoder (DAE) model for the ADS-B protocol, which effectively addresses the problem of identifying abnormal behaviors in multivariate time series data. Laine et al. [6] applied a multi-instance learning framework and combined it with a multihead convolutional neural network–recurrent neural network (MHCNN–RNN) architecture to predict precursors that may result in safety incidents during flight, improving the ability to identify potential risks. Hassan and Granelli [7] proposed the multi-layer Long Short-Term Memory (LSTM) network, which performance outperforms all other deep learning models across the entire range of tested parameters.

In terms of accident risk assessment, the two-dimensional cloud model constructed by Zhang and Shi [8] significantly improved the evaluation accuracy of the approach control system; the team led by Wu et al. [9] realized the risk prediction of key passengers via the improved XGBoost algorithm, XGBoost (extreme gradient boosting), which is an ensemble learning algorithm based on gradient-boosted decision trees that can improve the predictive performance of the model by integrating multiple weak evaluators. In the direction of trend prediction, the SVM–DNN hybrid model constructed by Zhang and Mahadevan [10] has been successfully applied to ASRS data analysis, and Srinivasan et al. [11] investigated the aviation accident prediction method based on LSTM, which uses word embedding and sequence modeling to predict accidents, aircraft damage and casualties, increasing the accuracy of accident prediction.

Recently, research on civil aviation safety has continued to expand, showing significant trends in intelligent methodologies, multimodal data, and forward-looking goals: At the level of analytical methods, traditional manual judgment is gradually shifting to intelligent analysis, and research data have expanded from single structured data to multimodal data; the focus of research has also shifted from passive accident tracing to active risk early warning. The in-depth integration of text mining and machine learning technologies has provided critical support for improving civil aviation safety levels. However, existing methods still face challenges, such as insufficient semantic understanding and difficulty in risk quantification in the field of cabin safety risk assessment. In this study, a deep learning model based on the CNN–LSTM–attention hybrid architecture is proposed. Through the synergistic effect of local feature extraction, temporal dynamic modeling, and attention mechanism optimization, the architecture achieves high-level semantic parsing of cabin safety texts and accurate quantification of risks. This research aims to overcome the limitations of traditional safety assessment, provide a new paradigm for building an active defense-oriented civil aviation safety system, and help safety management in the industry develop in depth toward digitization and intelligence.

The main contributions of this study are summarized as follows: (1) We propose an integrated framework of “deep text mining–intelligent risk assessment” for cabin safety incident analysis, combining text feature extraction and risk-level interpretation. (2) We develop a text mining pipeline using a bag-of-words (BOW) representation and chi-square feature selection to identify key risk-related terms from ASRS cabin safety narratives. (3) We construct a hybrid CNN-LSTM-Attention model, where Word2Vec embeddings provide distributed semantic representations, CNN captures local n-gram patterns, LSTM models long-range contextual dependencies, and additive attention highlights the most informative text segments for risk classification. (4) We establish a five-level risk grading scheme by mapping 34 event outcomes into five risk levels, enabling more interpretable risk quantification and mitigating class imbalance. (5) We conduct comparative experiments against baseline methods and evaluate performance using standard metrics, demonstrating the effectiveness of the proposed approach.

The remainder of this paper is organized as follows. Section 2 reviews the related literature on cabin safety management and text mining methods in aviation safety studies. Section 3 presents the proposed cabin safety risk assessment framework and introduces the CNN-LSTM-Attention model, including the text mining procedure and model components. Section 4 describes the data source and preprocessing, the risk-level grading strategy, and the experimental evaluation and comparative results. Finally, Section 5 concludes the study and discusses implications for cabin safety risk management and future research directions.

## 2 Literature review

As an important aspect of civil aviation safety, cabin safety involves the safety management of aircraft, pilots, cabin crews and passengers in a specific environment, and its safety level directly affects the overall safety level of civil aviation. When cabin safety is at risk, a series of safety incidents, such as emergency landing, diversion, reorientation and return to the departure airport, may occur, greatly affecting aviation safety. Therefore, cabin safety should not be treated in isolation but should be addressed from a higher level with a broader perspective and a system safety mindset. Research on civil aviation cabin safety has attracted much attention, and the literature related to this study can be roughly divided into aspects such as civil aviation safety optimization, civil aviation management strategies, civil aviation human factors, and civil aviation system research.

### 2.1 Optimization of civil aviation cabin safety

Currently, researchers are focusing on improving the ability to identify cabin safety risks through advanced technologies, as indicated below. Berkhahn [12] approached civil aviation cabin safety from the perspective of “data communication architecture for safety-critical cabin systems” and proposed, for the first time, a communication architecture featuring “triple redundancy + real-time fault self-healing.” This approach expanded the boundary of cabin safety from “single-point equipment reliability” to “system-level communication resilience”. Hsu and Liu [13] integrated “man–machine–environment–management–task” system factors through the five-M model, filling the gap in the systematic theoretical framework for cabin safety research. Zhang et al. [14] investigated evacuation efficiency in fire scenarios and proposed a design scheme to optimize cabin layout. Du et al. [15] established an emergency evacuation model for cabin passengers on the basis of cellular automaton theory, providing auxiliary data for the airworthiness certification of ARJ regional aircraft. Through research on the design of model-based complex system architectures in aircraft cabins, Ghanjaoud et al. [16] revealed via simulation results that the three key nodes in cabin illegal interference incidents are “cabin structure damage”, “insufficient airline training,” and “failure of airport police to promptly take over unruly passengers”. Čavka et al. [17] proposed the first “energy efficiency–safety” dual-objective design framework and introduced the “safety–energy efficiency index (SEI)” for multiobjective optimization of cabin design. Liu and Tong [18] constructed a cabin safety evaluation index system from six aspects—personnel, machinery, environment, management, information, and loading—via system engineering theory and conducted an empirical analysis. Cui et al. [19] established a response lag model for smoke detectors, providing theoretical support for optimizing the layout of cabin smoke detection equipment. Through theoretical models combined with engineering measurements, Belland et al. [20] systematically explored the safety and effectiveness boundaries of ultraviolet-C (UV-C) in cabins, promoting aviation epidemic prevention from “emergency response” to “active defense”. Wu et al. [21] developed a VR-based cabin lithium battery fire drill platform, and their research revealed that VR technology can effectively compensate for the shortcomings of traditional drills and increase practical training effects. Ling et al. [22] proposed an automatic cable segmentation method on the basis of knowledge distillation and context fusion, increasing the accuracy of cabin cable identification and providing technical support for preventing electrical faults and fire risks.

### 2.2 Civil aviation cabin safety management strategies

Studies have pointed out that comprehensive design and a sound safety management system are critical to increasing cabin safety, and different scholars have explored this topic from multiple dimensions: Molesworth and Burgess [23] approached the macro system of civil aviation cabin safety through “the effectiveness of auditory information,” prompting the industry to shift from “controlling equipment” to “empowering users” and providing an innovative “technology–policy–experience” trinity idea for cabin safety management. On the basis of the theory of planned behavior, Cui et al. [24] reported that risk perception, attitude, and behavioral control ability significantly affect the behavioral intentions of passengers and suggested the implementation of targeted safety education to reduce unsafe behavior. With a complex network model, Wang et al. [25] reported that controlling critical nodes such as crew members and the environment can reduce the probability of risk transmission and improve management levels. Çevik et al. [26] revealed the disregarded safety blind spot from “the uniqueness of cabin crew fatigue”: As pilot fatigue management becomes increasingly standardized, cabin crew fatigue is emerging as a new risk source. By quantifying the transmission mechanism of fatigue in service-safety operations, a “data-driven intervention plan” for cabin safety management can be provided. Through evolutionary game analysis, Wu et al. [27] clarified the decisive role of the safety disposal capabilities of airlines, government supervision, and the punishment intensity of airport public security in the emergency disposal effect of cabin disturbance incidents.

### 2.3 Aspects of civil aviation cabin personnel management

This research focuses on the effects of crew behavior and psychological factors on cabin safety, highlighting the importance of personnel management and training. For example, Zhang et al. [28] used a data-driven Bayesian network approach to conduct a risk analysis of civil aviation pilot factors from 2008 to 2020. Studies have shown that weather conditions and flight phases are relatively strongly correlated with the type of casualty in accidents, while human factors such as pilot decision-making are more likely to result in fatal accidents. Abdelhakim et al. [29] confirmed the necessity of food safety training from the “training–behavior safety” chain for the first time, expanding the boundary of cabin safety from traditional “equipment/emergency” to “daily operation compliance”. Kim et al. [30] reported that physical fatigue, psychological pressure, and complacency are the main causes of human errors among flight attendants and called for strengthening psychological and behavioral management. Through association rules and social network analysis, Liu et al. [31] reported that human factors account for the greatest proportion of the causes of cabin safety accidents, emphasizing that personnel management and training should be the focus.

### 2.4 Aspects of civil aviation system optimization

Currently, research on the causal analysis of aviation accidents is trending from single-factor identification to multidimensional and systematic analysis. In terms of research on causal systems, Guo et al. [32] applied the HFACS model combined with deep learning methods to analyze the causes of accidents during the approach and landing phases and clearly noted the key roles of human error, equipment failure, and organizational management defects in accidents. Yue and Li [33] constructed a causal analysis model of aviation accidents on the basis of reverse fuzzy Petri nets and identified the key factors in a man–machine–environment–management system through complex network analysis of 257 aviation accident investigation reports, providing new ideas for accident prevention and control. In terms of the application of text mining, Hu et al. [34] proposed a text mining method on the basis of an SVM and a DNN, which is used to classify NTSB accident reports and extract the text features of accident causes. Tanguy et al. [35] explored the aviation safety report analysis method based on NLP and proposed a complete set of tools for text classification, topic modeling, and similarity search, which can help aviation safety experts analyze accident reports efficiently.

Existing studies have shown that text mining technology has made considerable progress in the field of aviation safety. The integration of natural language processing, machine learning, and deep learning methods can effectively extract potential information from aviation accident reports, significantly increasing the accuracy of accident prediction and the efficiency of risk management and control. However, current research focuses mostly on traditional areas such as flight safety, air traffic control systems, and aircraft maintenance. Relatively few text mining studies target civil aviation cabin safety, and the application of related technologies is relatively backward. Existing cabin safety research focuses mainly on the macro level, such as the optimization of safety management systems and the improvement of operating procedures.

In this study, deep learning technology is applied to the risk management of civil aviation cabin safety to realize the automatic identification and accurate classification of cabin safety accidents and predict potential risk factors. Two innovative breakthroughs are achieved: From a research perspective, it is the first time that deep learning has been applied to the microrisk assessment of cabin safety; from a technical perspective, a full-process solution of “semantic understanding-intelligence assessment is constructed.” To provide a structured overview, Table 1 summarizes representative studies.

**Table 1:** Summary of representative studies on aviation safety text mining and risk assessment.

Studies	Domain&dataset	Technique(s)	Key strengths	Limitations / gaps
Rose et al. [1]	ASRS narratives	NLP clustering+K-means+t-SNE	Identifies latent patterns and trends from narratives	Mainly descriptive; limited risk quantification and prediction
Kuhn [2]	ASRS narratives	Structural topic modeling (STM)	Discovers latent topics without manual labels	Topic discovery may not directly support incident severity/risk classification
Zhao et al. [3]	Aviation accident reports	Hierarchical multilabel classification	Fine-grained event extraction and structured labeling	Requires careful annotation/design; may be complex for operational deployment
Hassan & Granelli [4]	Wireless/aviation-related channel data	1D-CNN	Strong feature extraction for sequential signals	Not focused on aviation safety text narratives
Chevrot et al. [5]	ADS-B / time-series	Discriminative autoencoder (DAE)	Effective anomaly detection in multivariate time series	Limited interpretability; not directly applicable to cabin safety narratives
Laine et al. [6]	Flight-related safety events	Multi-instance learning + MHCNN–RNN	Predicts precursors and captures temporal dependencies	Requires carefully curated instances; complexity increases with scale
Zhang & Mahadevan [10]	Aviation incident risk prediction	Ensemble ML (SVM–DNN hybrid)	Improved predictive performance by combining models	Feature engineering required; weaker semantic understanding than deep NLP models
Srinivasan et al. [11]	Aviation accident reports	LSTM + word embedding	Learns sequential representations for prediction	May miss local patterns; limited interpretability without attention mechanisms
Hu et al. [34]	NTSB accident reports	SVM + DNN	Effective text classification and indicator extraction	Typically needs feature selection; limited risk-level mapping
Tanguy et al. [35]	Aviation safety reports	NLP toolkit (classification, topic modeling, similarity search)	Comprehensive toolset supporting efficient expert analysis	Primarily analytic tools; lacks an integrated risk-level assessment framework

This table presents a structured overview of representative studies on aviation safety text mining and risk assessment, summarizing their research domain, techniques used, key strengths, and limitations or gaps.

Overall, existing studies demonstrate the value of text mining and machine learning for aviation safety analysis; however, most work focuses on general flight safety domains (e.g., ASRS or NTSB reports) and emphasizes clustering or prediction rather than interpretable risk quantification. Moreover, relatively limited attention has been paid to cabin-specific safety narratives and the challenges of imbalanced outcome distributions. These gaps motivate the integrated framework and the CNN–LSTM–Attention-based risk assessment approach proposed in this study.

## 3 Civil aviation cabin safety risk assessment model based on CNN-LSTM-attention

First, text mining is performed on the unstructured data in cabin safety accident reports via methods such as the bag-of-words model and chi-square statistics to extract relevant factors that may cause cabin safety accidents. Furthermore, according to the risk source identification and risk analysis comprehensive evaluation list, these risk factors are converted to risk sources with distinct causal mechanisms. Second, with a large amount of cabin safety incident data, an attention mechanism is introduced on the basis of CNN-LSTM to construct a CNN–LSTM–Attention hybrid model to improve the ability to dynamically capture key civil aviation cabin safety features.

### 3.1 Methods for identifying risk sources of civil aviation cabin safety

In this paper, text mining technologies (a bag-of-words model and chi-square dimensionality reduction) are used to automatically extract key causal features from accident reports, addressing the problems of time-consuming manual processing and insufficient coverage. Furthermore, combined with the hazard source identification and risk analysis comprehensive evaluation list of the cabin service department, risk factors are converted to risk sources with distinct causal mechanisms.

### 3.2 Ethics statement

This study does not involve human participants, animal experiments, or other content requiring ethical review. The research is conducted in strict accordance with academic integrity and ethical norms, and thus no ethical approval was required.

### 3.3 Bag-of-words model

In the field of text mining and safety analysis, the bag-of-words (BOW) model is widely used for extracting text feature items because of its simplicity and efficiency. By counting the number high-frequency words in cabin safety accident reports, this model can effectively identify critical safety risk factors. Compared with complex grammatical structure analysis, keyword frequency statistics are more explainable and focus on the most representative causal features.

Assume that there is a vocabulary *n* (containing all unique words in the documents) in the cabin safety accident report database. Each word in text *D* corresponds to a feature value according to its position in the vocabulary. Each document *D*_*i*_ can be represented as a feature vector *x*_*i*_, where each dimension corresponds to a word in the vocabulary, and its value represents the number of times the word appears in the document, as shown in Equation (1).


$$\begin{array}{c} x_{i}=(f_{1},f_{2},\ldots ,f_{n}) \end{array}$$ (1)

where *f*_*j*_ represents the occurrence frequency of the *j*-th word in the vocabulary in document *D*_*i*_.

### 3.4 Chi-square statistic

Some high-frequency words extracted by the bag-of-words model are used as the initial feature items of the text. Owing to the high dimension of these feature items and the inclusion of a large amount of redundant information, which reduces computational efficiency, dimension reduction on the text feature items of aviation safety accident reports needs to be performed. In the text feature dimension reduction step, this study uses the chi-square statistic to conduct a thorough screening of risk keywords. This method quantifies the strength of the correlation between words and risk categories by calculating the degree of deviation between the actual observed frequency and the theoretical expected frequency of specific words in accident reports. The statistic is calculated by Equation (2).


$$\begin{array}{c} \chi^{2}(t,c_{i})=\frac{n(ad-cd{)}^{2}}{\left(a+c)(b+d)(a+b)(c+d\right)} \end{array}$$ (2)

where *n* represents the total number of texts; *a* is the frequency of texts that belong to category *c*_*i*_ and contain the feature term *t*; *b* denotes the frequency of tex*t*s that do not belong to category *c*_*i*_ but contain the feature term *t*; *C* indicates the frequency of texts that belong to ca*t*egory *c*_*i*_ but do not contain the feature term *t*; and *d* represents the frequency of texts that neither belong to category *c*_*i*_ nor contain the fea*t*ure term *t*.

The $\chi^{2}$ value of the entire corpus can be calculated using Formula (3).


$$\begin{array}{c} \chi_{max}^{2}(t)=\max_{i=1}^{m}\{\chi^{2}(t,c_{i})\} \end{array}$$ (3)

By setting the threshold value *m*, many meaningless feature items can be eliminated, achieving effective dimension reduction of feature items. This method can significantly improve the representation of text features while reducing computational complexity.

### 3.5 Civil aviation cabin safety risk level assessment model

**Fig 1:**
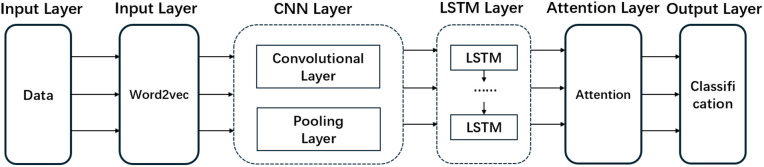
Network structure diagram of CNN–LSTM–attention model. The model processes input text through sequential layers: (1) an Embedding Layer using Word2Vec to create dense vector representations; (2) a CNN Layer with convolution and pooling to extract local features; (3) an LSTM Layer to capture long-range sequential dependencies; (4) an Attention Layer to weight important features; and (5) an Output Layer for final risk classification.

To accurately assess the risk of civil aviation cabin safety incidents, after the influencing factors of civil aviation cabin safety risks are identified, a large amount of cabin safety incident data is used to construct a civil aviation cabin safety risk level assessment model based on CNN–LSTM attention. The model uses mainly a hierarchical feature coding architecture, and the overall architecture of the model is shown in Fig 1. The main idea is as follows: first, the original text is mapped into high-dimensional dense vectors through the word embedding layer to realize the distributed representation of semantic information; second, the multiscale CNN module is used to extract local semantic patterns, and feature dimension reduction and key feature selection are achieved through pooling operations; third, the LSTM network is used to perform temporal modeling on feature sequences, and the long-distance contextual dependencies are adaptively captured through the gating mechanism; last, the attention mechanism is used to dynamically generate attention weights according to different semantic subspaces, which are fused to create a discriminative feature representation. As shown in Fig 1.

### 3.6 CNN module

(1) Convolutional layer The core operation of the convolutional layer is convolution, whose purpose is to extract features from local regions via convolution kernels (filters). Given an input text sequence, each token is first mapped to a d-dimensional embedding. The resulting input to the CNN can be represented as a matrix of size *L* × *d*, where *L* denotes the sequence length and *d* denotes the embedding dimension. We apply one-dimensional convolution along the sequence dimension, with convolutional filters spanning *k* consecutive tokens and the full embedding dimension. This design allows the model to capture local n-gram features in the text sequence. The output *c*_*i*_ of the convolution operation can be expressed as Equation (4).
$$\begin{array}{c} Z(i,j)=\sum_{p=0}^{k-1}\sum_{q=0}^{k-1}W(p,q)\cdot X(i+p,j+q)+b \end{array}$$ (4)
  The convolution result *c*_*i*_ is subsequently subjected to a nonlinear transformation through the activation function f(·) to obtain the output *a*_*i*_ of the convolutional layer, which can be expressed as Formula (5).
$$\begin{array}{c} A(i,j)=f\left(Z(i,j)\right) \end{array}$$ (5)
(2) Max pooling Max pooling is applied along the sequence dimension to aggregate the most salient features across different token positions, and its main functions are described as follows: First, max pooling reduces the spatial dimension of the feature maps through a sliding window mechanism, significantly decreasing the computational complexity of the model; second, max pooling selectively retains the strongest activation features in local regions, improving the ability of the model to identify key features; and last, the generalization performance of the model is improved by introducing translation invariance.
  Compared with average pooling, max pooling is more suitable for text processing tasks. Text features are often characterized by sparsity and local significance, and this method can more effectively capture discriminative key semantic units. The specific calculation process can be formalized as Equation (6), where max pooling is applied over the local feature regions to generate a condensed representation that is passed to subsequent layers.
$$\begin{array}{c} A_{pool}(i,j)=\max_{p,q\in \text{pool window}}A(i+p,j+q) \end{array}$$ (6)
(3) Fully connected layer
  After several layers of convolution and pooling operations, the resulting multidimensional feature maps are flattened into a one-dimensional vector and input into the fully connected layer. The fully connected layer performs a linear transformation to map the features to the final classification space. Let the input vector of the fully connected layer be *x*, the weight matrix be *W*, and the bias be *b*. Afterward, the output after calculation can be formalized as Equation (7).
$$\begin{array}{c} y=W_{fc}\cdot a+b_{fc} \end{array}$$ (7)

### 3.7 LSTM layer

The LSTM network is composed mainly of three main gating units—the input gate, forget gate and output gate—and includes a candidate memory unit and current memory unit. The specific calculation formulas are presented as follows:

(1) Input gate: This gating unit is used to determine how much information from the current input *x*_*t*_ needs to be written into the memory unit, and its calculation process can be formalized as Formula (8).
$$\begin{array}{c} i_{t}=\sigma \left(W_{i}x_{t}+U_{i}h_{t-1}+b_{i}\right) \end{array}$$ (8)
(2) Forget gate: This gating unit is used to determine how much information in the memory cell *C*_*t*−1_ at the previous moment needs to be retained, and its calculation process can be formalized as Equation (9).
$$\begin{array}{c} f_{t}=\sigma \left(W_{f}x_{t}+U_{f}h_{t-1}+b_{f}\right) \end{array}$$ (9)
(3) Candidate memory cell: This gating unit is used to generate new candidate information, which is combined with the input gate to determine whether it should be added to the memory cell. The calculation process can be formalized as Equation (10):
$$\begin{array}{c} {\tilde{C}}_{t}=\tanh\left(W_{c}x_{t}+U_{c}h_{t-1}+b_{c}\right) \end{array}$$ (10)
(4) Memory cell update: The memory cell is updated by combining the forget gate and the input gate, and its calculation process can be formalized as Equation (11):
$$\begin{array}{c} C_{t}=f_{t}⊙C_{t-1}+i_{t}⊙{\tilde{C}}_{t} \end{array}$$ (11)
  where ⊙ denotes the element-wise (Hadamard) product. (5) Output gate: This gating unit determines how much information in the current memory cell *C*_*t*_ is output to the next moment, and its calculation process can be formalized as Equation (12):
$$\begin{array}{c} o_{t}=\sigma \left(W_{o}x_{t}+U_{o}h_{t-1}+b_{o}\right) \end{array}$$ (12)
(6) Hidden state: The final output hidden state *h*_*t*_ is jointly determined by the output gate and the current memory cell, and its calculation process can be formalized as Equation (13):
$$\begin{array}{c} h_{t}=o_{t}⊙\tanh(C_{t}) \end{array}$$ (13)
  In the above formulas, *x*_*t*_ represents the current input; *h*_*t*−1_ represents the hidden state at the previous moment; *C*_*t*−1_ denotes the memory cell at the previous moment; *W* and *U* indicate the input weight matrix and the hidden state weight matrix, respectively; *b* represents the bias term; σ denotes the sigmoid activation function, whose output range is [0, 1] and is used to control the flow of information; and tanh indicates the hyperbolic tangent activation function, whose output range is [−1, 1].

### 3.8 Attention layer

In the text reports of cabin safety accidents, different paragraphs and sentences vary in their importance. The attention mechanism can dynamically assign weights to each time segment of the text sequence to highlight key information. In this study, an additive attention mechanism is used to model the text reports of cabin safety accidents. With trainable parameters, this mechanism dynamically distributes attention weights to different text segments. Compared with the dot-product attention mechanism, the additive attention mechanism is more suitable for scenarios involving long texts with unevenly distributed importance. This mechanism can effectively capture important causal information and nonlinear relationships in accident reports while increasing classification accuracy and improving the explainability of the model. Its calculation process can be formalized as Equations (14)–(16):


$$\begin{array}{c} e_{t}=\tanh(W_{s}h_{t}+b_{s}) \end{array}$$ (14)

$$\begin{array}{c} \alpha_{t}=\frac{\exp(e_{t})}{\sum_{j}\exp(e_{j})} \end{array}$$ (15)

$$\begin{array}{c} c=\sum_{t}\alpha_{t}h_{t} \end{array}$$ (16)

Where *h*_*t*_ represents the output hidden state vector of the LSTM layer at time step *W*_*s*_ and *b*_*s*_ represent the trainable parameters. The attention weight $\alpha_{t}$ is calculated, and then the context feature vector *c* is obtained through weighted summation.

### 3.9 Output layer

The output layer uses the softmax function to convert the output of the fully connected layer to a probability distribution of various categories, and its calculation process can be formalized as Equation (17):


$$\begin{array}{c} {\hat{y}}_{k}=\frac{\exp(y_{k})}{\sum_{j}\exp(y_{j})} \end{array}$$ (17)

## 4 Case analysis

### 4.1 Model training and evaluation details

To ensure the reproducibility of the proposed CNN–LSTM–Attention model, the main training and testing settings are described as follows.

Text preprocessing included tokenization and lowercasing. Stop words were not removed, as neural models with word embeddings can learn to down-weight non-informative tokens automatically.

For the deep learning models (CNN, CNN–LSTM, and CNN–LSTM–Attention), each token was mapped to a dense vector using Word2Vec embeddings. The embedding dimension was set to 300. All sequences were truncated or padded to a maximum length of 1000 tokens before being fed into the models.

To address the severe class imbalance among the 34 event outcomes, the original event categories were remapped into five risk levels (high, medium-high, moderate, medium-low, and low risk), and this risk-level stratification was used during model training and evaluation.

The dataset was split into training and test sets with a ratio of 70% to 30% using a stratified split to preserve the distribution of risk levels.

### 4.2 Data sources and processing

#### 4.2.1 Data sources.

The experimental data were obtained from the online database of the Aviation Safety Reporting System (ASRS) at the National Aeronautics and Space Administration (NASA) Center. After filtering through the event assessment and date filters, 12,967 abnormal aviation events in civil aviation cabins from 2004 to 2024 were selected. After cleaning, 8,280 valid text data points were retained, encompassing 34 independent outcomes, as listed in Table 2.

**Table 2:** Description of event outcomes.

Number	Outcome
x1	Flight crew overcame equipment problem
x2	Flight crew landed under emergency conditions
x3	Flight crew diverted
x4	Flight crew took evasive action
x5	Flight crew became reoriented
x6	Flight crew returned to departure airport
x7	Flight crew landed as a precaution
x8	Flight crew aborted takeoff
x9	Flight crew regained aircraft control
x10	Flight crew inflight shutdown
x11	Flight crew returned to gate
x12	Flight crew requested ATC (Air Traffic Control) assistance clarification
x13	Flight crew returned to clearance
x14	Flight crew executed go around missed approach
x15	Flight crew FLC (Flight Level Change) overrode automation
x16	Flight crew FLC complied automation with advisory
x17	Flight crew overrode automation
x18	Flight crew exited penetrated airspace
x19	Aircraft damaged
x20	Aircraft equipment problem dissipated
x21	Aircraft automation overrode flight crew
x22	Air traffic control issued new clearance
x23	Air traffic control issued advisory/alert
x24	Air traffic control provided assistance
x25	Air traffic control separated traffic
x26	General none reported taken
x27	General declared emergency
x28	General Physical Injury / Incapacitation
x29	General Maintenance Action
x30	General Evacuated
x31	General Work Refused
x32	General Flight Cancelled Delayed
x33	General Police Security Involved
x34	General release refused Aircraft not accepted

This table lists the 34 independent event outcomes extracted from the Aviation Safety Reporting System (ASRS) database, each labeled from x1 to x34, including outcomes such as flight crew diverted, aircraft damaged, general declared emergency, and general police security involved.

### 4.3 Imbalance handling

**Fig 2:**
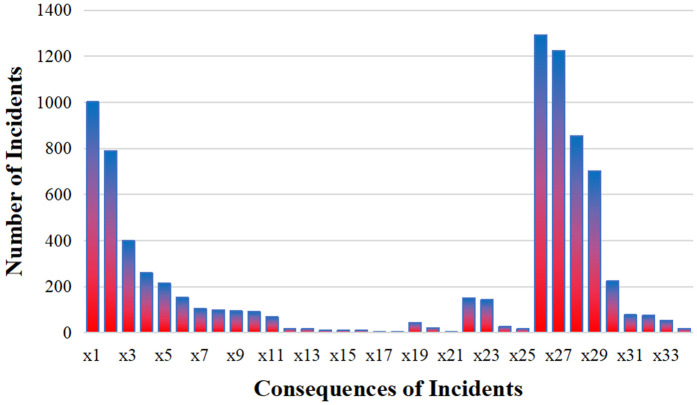
Distribution of civil aviation cabin event outcomes. The histogram displays the frequency of each outcome (x1 to x34) in the dataset (N = 8,280). The highly imbalanced distribution, with some outcomes occurring much more frequently than others, motivates the five-level risk stratification used in this study. For brevity, only outcomes with non-zero incident counts are shown.

The number of records for one outcome in the data report may be significantly greater than that of other categories. The distribution of outcomes for all hazardous events reported between 2004 and 2024 is shown in Fig 2.

Owing to the significant class imbalance in cabin safety incident records, while traditional machine learning algorithms are usually based on the assumption of balanced data, such data skew severely affects model performance. Therefore, in this study, in accordance with international aviation safety classification standards, the incident consequences are divided into five-level risk systems (high risk, medium–high risk, moderate risk, medium–low risk, and low risk). The data distribution is reconstructed through a structured risk grading strategy to improve the ability of the model to identify key high-risk incidents (see Table 3).

**Table 3:** Correspondence table of event outcome risk levels.

Risk Level	Outcome
High risk	General declared emergency
High risk	General physical injury/incapacitation
High risk	Flight crew inflight shutdown
High risk	Air traffic control separated traffic
High risk	Aircraft damaged
Medium–high risk	General evacuation
Medium–high risk	Flight crew regained aircraft control
Medium–high risk	Air traffic control issued advisory/alert
Medium–high risk	Flight crew landed under emergency conditions
Moderate risk	General work refused
Moderate risk	Flight crew became reoriented
Moderate risk	Flight crew diverted
Moderate risk	Flight crew executed go around missed approach
Moderate risk	Flight crew overcame equipment problem
Moderate risk	Flight crew rejected takeoff
Moderate risk	Flight crew took evasive action
Moderate risk	Air traffic control issued new clearance
Medium–low risk	General maintenance action
Medium–low risk	General flight cancelled delayed
Medium–low risk	General release refused aircraft not accepted
Medium–low risk	Flight crew overrode automation
Medium–low risk	Flight crew FLC overrode automation
Medium–low risk	Flight crew exited penetrated airspace
Medium–low risk	Flight crew requested ATC assistance clarification
Medium–low risk	Flight crew landed as a precaution
Medium–low risk	Flight crew returned to clearance
Medium–low risk	Flight crew returned to departure airport
Medium–low risk	Aircraft automation overrode flight crew
Low risk	General police security involved
Low risk	Flight crew returned to gate
Low risk	Aircraft equipment problem dissipated
Low risk	Air traffic control provided assistance
Low risk	General none reported taken
Low risk	Flight crew FLC complied automation with advisory

This table maps the 34 original event outcomes into a five-level risk system (high risk, medium-high risk, moderate risk, medium-low risk, and low risk) according to international aviation safety classification standards to address class imbalance.

**Fig 3:**
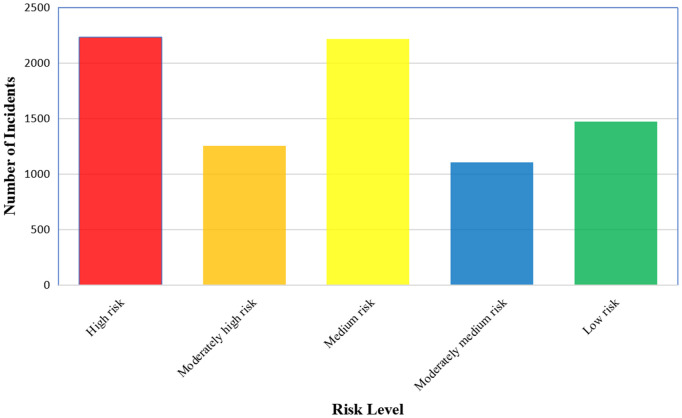
Distribution of risk levels. The bar chart illustrates the proportion of each risk category—High, Medium-High, Moderate, Medium-Low, and Low—after regrouping the 34 original event outcomes (N = 8,280). This stratification yields a more balanced distribution compared to the original data, mitigating class imbalance for model training.

To address the issue of highly imbalanced distribution among the 34 types of events in the original data, this study remaps 8,280 abnormal aviation events through a five-level risk stratification strategy. As shown in Fig 3, the risk level classification effectively improves the distribution of event categories to achieve a more balanced distribution. Ensuring the integrity of the main body is conducive to increasing the accuracy of the model.

### 4.4 Model validation

#### 4.4.1 Evaluation indicators.

In the multiclass classification task involving accident-causing factors and accident risk levels, to comprehensively assess the classification performance of the constructed model, the accuracy, precision, recall, and F1 score are selected as evaluation indicators. The structure of the confusion matrix introduced first is shown in Table 4.

**Table 4:** Structure of the confusion matrix.

	Predicted as Positive	Predicted as Negative
**Actually Positive**	True Positive (TP)	True Negative (TN)
**Actually Negative**	False Positive (FP)	False Negative (FN)

This table presents the basic structure of a confusion matrix for multiclass classification tasks, defining the four categories: True Positive (TP), True Negative (TN), False Positive (FP), and False Negative (FN).

TP (true positive) samples predicted as positive that are actually positive. TN(True Negative) refers to samples that are predicted as negative and are actually negative. FP (false positive) samples predicted as positive that are actually negative. FN (false negative) samples predicted as negative that are actually positive. The accuracy, precision, recall, and F1 score are calculated through the confusion matrix, with the calculation formulas shown in Table 5.

**Table 5:** Evaluation indicators.

Evaluation Indicators	Meaning	Calculation Formula
Accuracy	The proportion of correctly predicted samples to the total number of samples. It serves as a measure of the overall prediction accuracy of the model.	$\frac{TP+TN}{TP+TN+FP+FN}$
Precision	Among the samples predicted as positive by the model, the proportion that are actually positive. It measures the ‟cleanliness” of the predictions, specifically, how low the proportion of false positives is.	$\frac{TP}{TP+FP}$
Recall	Among all samples that are actually positive, the proportion that is correctly identified by the model. It focuses on the ability of the model to capture positive instances and includes instances of missed detections, or false negatives (FNs).	$\frac{TP}{TP+FN}$
F1 score	The harmonic mean of precision and recall serves as a comprehensive measure of both the accuracy and coverage of the model’s predictions for positive instances. In situations where there is a trade-off between precision and recall, the F1 score is a balanced evaluation metric.	$2\times \frac{Precision\times Recall}{Precision+Recall}$

This table defines the four evaluation metrics used in this study—Accuracy, Precision, Recall, and F1 score—along with their meanings and corresponding calculation formulas based on the confusion matrix.

### 4.5 Comparative analysis results

**Fig 4:**
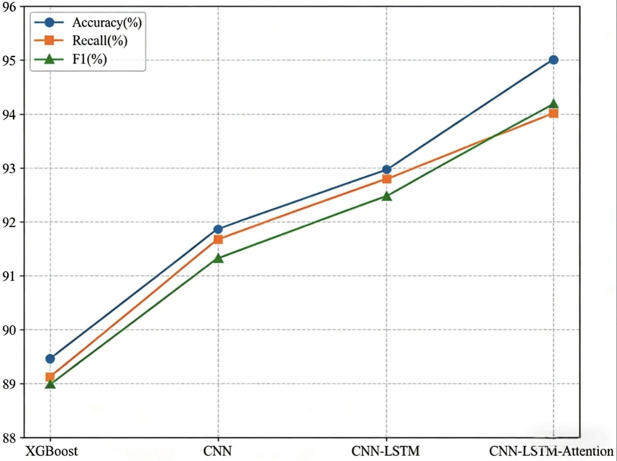
Comparison chart of model evaluation indicators based on the test set. The line chart displays the Accuracy, Recall, and F1-score for four models: XGBoost, CNN, CNN–LSTM, and the proposed CNN–LSTM–Attention. The proposed model achieves the highest scores across all three metrics (Accuracy, Recall, F1-score), demonstrating its superior capability in classifying cabin safety incident risk levels compared to baseline approaches.

In this study, 8,280 aviation anomaly events recorded between 2004 and 2024 were randomly divided into a training set and a test set at a ratio of 7:3. Specifically, 5,796 events (70%) were allocated to model training, and 2,484 events (30%) were used to assess the generalizability of the model. The training set data were then input into various models, including XGBoost, CNN, CNN–LSTM, and CNN–LSTM–attention, for training and prediction purposes. Table 6 presents the training accuracy of these four models, and Table 7 outlines their prediction and evaluation metrics. To emphasize the performance comparison, the performance indicators of each model are shown in a line chart in Fig 4.

**Table 6:** Training accuracy of different models.

Models	Accuracy (%)
XGBoost	90.44
CNN	92.07
CNN–LSTM	93.50
CNN–LSTM–attention	95.63

This table compares the training accuracy of four models: XGBoost, CNN, CNN-LSTM, and the proposed CNN-LSTM-Attention model, demonstrating the superior training performance of the proposed approach.

**Table 7:** Comparison of model evaluation metrics based on the test set.

Models	Accuracy (%)	Recall (%)	F1 score (%)
XGBoost	89.45	89.12	88.98
CNN	91.87	91.68	91.33
CNN–LSTM	92.95	92.78	92.47
CNN–LSTM–Attention	95.01	94.01	94.17

This table presents the test set performance comparison across four models using Accuracy, Recall, and F1 score, showing that CNN-LSTM-Attention achieves the best results.

(1) Analysis of the training accuracy of the model
  As shown in Table 6, the CNN–LSTM–attention model has significant advantages in the cabin safety text analysis task. Its training accuracy of 95.63% not only far exceeds that of the traditional XGBOOST model (90.44%) but is also 3.56% and 2.13% higher than that of the CNN (92.07%) and CNN–LSTM (93.50%), respectively. This advantage stems from its multilevel feature extraction capability: the CNN layer effectively captures local text patterns, the LSTM layer models long-distance contextual dependencies, and the attention mechanism dynamically focuses on key information, thereby achieving accurate modeling of the deep semantics of the text.
(2) Analysis of the generalizability of the model
  As shown in Table 7 and Fig 4, the accuracy, recall, and F1 score of XGBoost on the test set are inferior to those of the deep learning models (CNN, CNN–LSTM, and CNN–LSTM–attention). Although XGBoost remains an efficient benchmark method because of its advantages of fast computing speed and strong interpretability, it is limited in its ability to extract deep semantic features. The CNN efficiently extracts local text features through convolution operations, and its classification performance on the test set surpasses that of XGBoost. CNN–LSTM uses an LSTM structure to model the temporal dependencies of text, and its accuracy and F1 score are significantly better than those of the CNN. The CNN–LSTM–attention model uses an attention mechanism on this basis, which can dynamically assign weights to focus on key information, achieving better classification results on the test set and demonstrating better generalizability.

The CNN–LSTM–attention model in this study achieved the best performance, demonstrating the significant advantages of deep learning methods that combine local feature extraction, sequence modeling, and attention mechanisms in the risk assessment of civil aviation cabin safety.

### 4.6 Level-wise performance and error analysis

To provide a more detailed evaluation, Table 8 reports precision, recall, and F1-score for each of the five risk levels. The proposed model achieves strong and stable performance across all levels, with F1-scores ranging from 93.93% to 96.73%. In particular, the high-risk level achieves a precision of 97.58%, a recall of 95.65%, and an F1-score of 96.60%, indicating that the model can effectively identify severe cabin safety incidents. The macro-averaged and weighted-averaged F1-scores are 95.75% and 95.99%, respectively, suggesting robust overall performance despite class imbalance.

**Table 8:** Level-wise performance for the five risk levels.

Risk level	Precision (%)	Recall (%)	F1 score (%)	Support (n)
High	97.58	95.65	96.60	1562
Medium–high	96.53	95.43	95.97	877
Moderate	97.69	95.78	96.73	1551
Medium–low	93.34	94.53	93.93	774
Low	93.60	97.48	95.50	1032
Macro avg	95.75	95.77	95.75	–
Weighted avg	96.18	95.83	95.99	–

This table reports detailed precision, recall, and F1-score for each of the five risk levels, demonstrating strong and stable performance across all levels with F1-scores.

Table 9 presents the confusion matrix (in sample counts) for the five-level risk classification task on the test set. Overall, correct predictions dominate the diagonal entries, showing strong discrimination among the five risk levels. For the high-risk category, 1494 out of 1562 cases are correctly classified, while the remaining errors are relatively small and mainly distributed across the other four levels (10 as moderately high risk, 21 as medium risk, 18 as moderately medium risk, and 19 as low risk). Similarly, the moderately high-risk and medium-risk categories show strong diagonal dominance (837/877 and 1475/1551 correct predictions, respectively).

**Table 9:** Confusion matrix for five-level risk classification.

	High risk	Moderately high risk	Medium risk	Moderately medium risk	Low risk
**High risk**	1494	10	21	18	19
**Moderately high risk**	4	837	4	13	19
**Medium risk**	18	14	1475	16	28
**Moderately medium risk**	6	5	9	742	12
**Low risk**	9	3	6	8	1006

This table presents the confusion matrix with sample counts for the five-level risk classification task. Rows indicate the true risk levels and columns indicate the predicted risk levels; values denote sample counts, showing strong diagonal dominance and accurate classification across all risk levels.

### 4.7 Risk assessment of civil aviation cabin safety

The texts related to risk sources in the test set are input into the constructed CNN–LSTM–attention model, and the predicted corresponding risk levels are shown in Table 10.

**Table 10:** Risk level classification table.

Risk Level	Feature Items	Risk Sources
High risk	Turbulence	Risk of personal injury caused by sudden turbulence during the flight
High risk	Smoke	Risk of panic caused by visible smoke in the cabin
High risk	Engine	Risk of abnormal engine vibration being transmitted to the cabin
High risk	Electrical	Risk of fire caused by a short circuit in the cabin electrical system
High risk	Burning smell	Risk of panic evacuation caused by unusual burning odors
High risk	Oxygen mask	Risk of oxygen mask falling off/insufficient oxygen supply
High risk	Hit head	Risk of injury due to head impact
High risk	QRH (Quick Reference Handbook)	Risk of incorrect use of the quick reference handbook
High risk	Medical emergency	Risk of treatment delay arising from insufficient emergency resources aboard the aircraft
High risk	Flight attendant	Risk associated with nonstandard emergency procedures conducted by flight attendants
Medium–high risk	Fume, fume event	Risk of poisoning due to fuel vapor entering the cabin
Medium–high risk	Passenger	Risk of loss of cabin order caused by passenger conflicts
Medium–high risk	Smell, odor	Risk of misjudgment and health hazards caused by unusual odors
Medium–high risk	Device	Risk of cabin equipment failure
Medium–high risk	Seat belt	Risk of passengers being thrown due to not wearing seat belts
Medium–high risk	Crew scheduling	Risk of fatigue caused by unreasonable crew scheduling systems
Low risk	Galley cart	Risk of impact due to failure of galley cart fixing devices
Low risk	Airline policy	Risk of response delay caused by loopholes in safety procedures

This table presents the risk level classification results for cabin safety risk sources identified from the test set, listing the risk level, corresponding feature items, and detailed descriptions of risk sources for high risk, medium-high risk, and low risk categories.

As shown in Table 10, the main aspects of high-risk accidents focus on the following key indicators: turbulence, smoke, electrical faults, burning smells, use of oxygen masks, head impact, QRH (calling of the quick reference handbook), medical emergency, abnormal engine, and related to flight attendant operations. All these factors pose direct and serious risks to aviation cabins and flight safety. For example, turbulence may cause personal injuries; smoke or smells may indicate fire risks; and engine or electrical faults may even have catastrophic consequences.

In contrast, medium- to high-risk factors such as gas leakage are slightly less harmful but still pose potential risks to health; medium-risk factors such as unusual smells, abnormal passenger behavior, and seat belt issues reflect more potential hidden dangers in operational management, whereas low-risk factors such as galley cart-related or policy implementation do not pose immediate risks but may increase risks in specific situations. Hierarchical risk distribution characteristics range from main system failures to operational management omissions.

## 5 Conclusion

In this paper, event information is extracted from the ASRS. In view of its characteristics, such as unstructured data and data imbalance, the ASRS quantifies and classifies the event risk levels, maps them, and designs a risk assessment model. This approach improves the classification and prediction accuracy of event summaries and provides support for proactive safety risk management in the cabin. The conclusions are as follows:

(1) According to actual risk management requirements, all event outcomes are divided into 5 levels, which not only facilitates the intuitive assessment of event risks from an engineering perspective but also addresses the problem of data imbalance at the algorithm level.
(2) A hybrid deep learning model based on CNN—LSTM–Attention is constructed to predict the risk levels of civil aviation cabin safety accidents. Experiments show that this model has significant advantages in the task of predicting the risk levels of civil aviation cabin safety incident texts, with a classification accuracy of more than 95% on the test set. This represents a substantial improvement over traditional models such as XGBoost and basic deep learning models. This model can effectively integrate the local features and global semantic information of cabin accident reports, highlight key content with an attention mechanism, and significantly reduce the misclassification of samples with adjacent risk levels and achieves higher accuracy than a single model does.
(3) The application of deep learning in the field of civil aviation cabin safety has significantly improved the capabilities of automatic identification and risk prediction for civil aviation cabin safety incidents, is important for improving civil aviation cabin safety risk management systems and provides an effective approach for exploring the digital transformation of civil aviation cabin safety management.

Although the proposed CNN–LSTM–Attention framework demonstrates strong performance for cabin safety risk classification, several directions remain for future research. First, future work will explore more advanced pre-trained language models (e.g., domain-adapted Transformer-based models) to further improve semantic understanding and robustness for long and complex incident narratives. Second, we will investigate multi-label learning to reflect the fact that a single cabin incident may involve multiple outcomes and risk factors. Third, incorporating additional structured or contextual information (e.g., flight phase, aircraft type, and operational conditions) together with narrative text may enable more accurate and interpretable risk assessment. Finally, we plan to develop a real-time or near-real-time decision-support prototype that can assist airlines and regulators in early warning and proactive cabin safety management.
